# Using computer, mobile and wearable technology enhanced interventions to reduce sedentary behaviour: a systematic review and meta-analysis

**DOI:** 10.1186/s12966-017-0561-4

**Published:** 2017-08-11

**Authors:** Aoife Stephenson, Suzanne M. McDonough, Marie H. Murphy, Chris D. Nugent, Jacqueline L. Mair

**Affiliations:** 1 Shore Rd, Newtownabbey, BT37 0QB Northern Ireland; 2 Centre for Health and Rehabilitation Technologies, Institute of Nursing and Health Research, Faculty of Life and Health Sciences, Ulster University, Shore Rd, Newtownabbey, BT37 0QB Northern Ireland; 3 UKCRC Centre of Excellence for Public Health (Northern Ireland), Belfast, Northern Ireland; 4 School of Physiotherapy, University of Otago, Dunedin, New Zealand; 5 Computer Science Research Institute, Faculty of Computing and Engineering, Ulster University, Shore Rd, Newtownabbey, BT37 0QB Northern Ireland

**Keywords:** Sedentary behaviour, Behaviour change, Randomised-controlled trials, Systematic review, Digital technology

## Abstract

**Background:**

High levels of sedentary behaviour (SB) are associated with negative health consequences. Technology enhanced solutions such as mobile applications, activity monitors, prompting software, texts, emails and websites are being harnessed to reduce SB. The aim of this paper is to evaluate the effectiveness of such technology enhanced interventions aimed at reducing SB in healthy adults and to examine the behaviour change techniques (BCTs) used.

**Methods:**

Five electronic databases were searched to identify randomised-controlled trials (RCTs), published up to June 2016. Interventions using computer, mobile or wearable technologies to facilitate a reduction in SB, using a measure of sedentary time as an outcome, were eligible for inclusion. Risk of bias was assessed using the Cochrane Collaboration’s tool and interventions were coded using the BCT Taxonomy (v1).

**Results:**

Meta-analysis of 15/17 RCTs suggested that computer, mobile and wearable technology tools resulted in a mean reduction of −41.28 min per day (min/day) of sitting time (95% CI -60.99, −21.58, I2 = 77%, *n* = 1402), in favour of the intervention group at end point follow-up. The pooled effects showed mean reductions at short (≤ 3 months), medium (>3 to 6 months), and long-term follow-up (>6 months) of −42.42 min/day, −37.23 min/day and −1.65 min/day, respectively. Overall, 16/17 studies were deemed as having a high or unclear risk of bias, and 1/17 was judged to be at a low risk of bias. A total of 46 BCTs (14 unique) were coded for the computer, mobile and wearable components of the interventions. The most frequently coded were “prompts and cues”, “self-monitoring of behaviour”, “social support (unspecified)” and “goal setting (behaviour)”.

**Conclusion:**

Interventions using computer, mobile and wearable technologies can be effective in reducing SB. Effectiveness appeared most prominent in the short-term and lessened over time. A range of BCTs have been implemented in these interventions. Future studies need to improve reporting of BCTs within interventions and address the methodological flaws identified within the review through the use of more rigorously controlled study designs with longer-term follow-ups, objective measures of SB and the incorporation of strategies to reduce attrition.

**Trial registration:**

The review protocol was registered with PROSPERO: CRD42016038187

**Electronic supplementary material:**

The online version of this article (doi:10.1186/s12966-017-0561-4) contains supplementary material, which is available to authorized users.

## Introduction

Sedentary behaviour (SB) has been defined as any waking behaviour characterised by energy expenditure of 1.5 metabolic equivalents (METs) or less, undertaken while in a sitting or reclining posture [1]. Modern society provides many opportunities for prolonged sitting in leisure, work and commuting [2]. Data from a range of industrialised countries suggest that SB is highly prevalent with the majority of people’s time (55–69% of the day) spent in sedentary pursuits [3–6].

Prolonged SB is positively associated with a range of health concerns including all-cause mortality, cardiovascular disease, type 2 diabetes, metabolic syndrome and several types of cancers [7]. Although the precise physiological mechanisms by which SB is detrimental to health are not fully known, a sedentary lifestyle is associated with cardiovascular morbidity and mortality, defects in lipoprotein metabolism, early atherosclerosis, insulin resistance, and development of the metabolic syndrome [2].

Previous systematic reviews and meta-analyses suggest that it is possible to intervene to reduce SBs in adults through activity permissive work stations, height adjustable desks, health coaching, activity monitors, and prompts to break up sitting [8, 9]. Pooled results from these interventions range from 22 to 91 min/day reduction in sedentary time in the intervention groups compared with the controls. While technological advancements have contributed to a rise in SB [10], these reviews [8, 9] have identified that they are also being harnessed to reduce SB. Digital tools such as mobile phones, internet, text-messaging and wearable sensors can provide a platform to intervene to change health behaviours, however, there is a lack of evidence examining their role in reducing SB. These have been successfully applied to improve diet/Physical Activity (PA) [11, 12], sexual health behaviours [13], weight management [14], alcohol reduction [12] and smoking cessation [15, 16]. One systematic review and meta-analysis investigated the use of mobile phone based interventions on outcomes of PA and SB [17]. The main findings were that these interventions targeting PA and SB promote small reductions in free-living individuals’ sitting time. However, only 5 of the 21 included studies reported a measure of SB.

Recent recommendations on prevention and management of non-communicable diseases stressed the need for research focused on behaviour change as the core component [18]. The identification and characterisation of behaviour change techniques (BCTs) allows for an understanding of mechanisms of behaviour change, leading to enhanced replication and implementation of effective interventions [19]. Whilst reviews of SB interventions and the BCTs used within these interventions have started to emerge, they are scarce and have lacked a clear aim to reduce SB exclusively [8, 9, 17, 20]. The effectiveness of interventions supported by computer, mobile and/or wearable technology aimed specifically at reducing SB, and the BCTs used within, have not yet been explored. The objectives of this review are to evaluate the effectiveness of behaviour change interventions using computer, mobile and/or wearable technologies aimed at reducing SB in healthy adults and to identify the BCTs used within these interventions.

## Methods

The Preferred Reporting Items for Systematic reviews and Meta-Analyses (PRISMA) Guidelines and Cochrane Handbook for Systematic Reviews of Interventions were used as a methodological template for this review [21, 22] (Additional file 1).

**Inclusion criteria**

- Adults aged 18 years and over,
- Published RCTs of any duration with a main aim of reducing SB and with computer, mobile or wearable technology as any part of the intervention,
- RCTs with a comparison or control arm that consisted of no intervention control, usual care, or alternative treatment conditions,
- Pre-post objective, subjective or proxy measure of SB.

**Exclusion criteria**

- RCTs not published in English,
- Comparator intervention using computer, mobile or wearable technology to reduce SB or increase PA,
- RCTs where the main aim of the intervention was to increase PA,
- Interventions delivered in a hospital setting,
- Clinically diagnosed populations, with the exception of those who are overweight or obese.

### Information sources and search strategy

Search strategies were developed for each electronic database; MEDLINE, EMBASE, CINAHL, PsycINFO and PubMed. The searches were based on the strategy developed for MEDLINE (Additional file 2) and revised appropriately for the other databases.

The search results were imported into EndNote X7 bibliographic software (Thompson Reuters, San Francisco, CA, USA) and duplicate studies were removed. The titles and abstracts of all identified studies were screened to identify potentially relevant papers. Studies that did not meet the inclusion criteria and titles/abstracts obviously not related to the topic of interest were excluded. Full text papers of potentially relevant studies were retrieved and assessed for eligibility by one member of the research team. Where uncertainties arose regarding study inclusion, consensus was achieved through discussion amongst the research team.

### Data extraction

The following data were independently extracted from each article using a standardised form: author, year, study design, participants, intervention description, comparator description, SB outcome measures and longest follow-up.

### Assessment of risk of bias in included studies

The risk of bias for each study was assessed using the Cochrane Collaboration’s risk of bias tool [22]. Initially, a small sample of studies (*n* = 3) were assessed by two members of the research team, inconsistency in scoring was reviewed, and a consensus reached prior to the analysis of the remaining studies, by one author. The remainder of the risk of bias assessment was carried out independently by one member of the research team.

Studies that used an objective measure to assess SB were judged as being at low risk of bias for blinding of outcome assessment. Studies assessing SB with subjective and proxy measures were judged as being at high risk of bias, as there was potential for misreporting of time spent sitting. Where greater than 20% dropout in any group for outcomes up to one year and greater than 30% for outcomes greater than one year was reported, studies were judged as being at high risk of bias for incomplete outcome data. Studies were judged as being at low risk of bias for selective outcome reporting if the final publication of the trial followed what had been planned in a published protocol paper. In the case where no protocol paper was publicly available, studies were deemed as being at low risk for selective outcome reporting if they had reported all the outcomes mentioned in the methodology. A study was judged to be at low risk of bias overall when all domains had a low risk of bias. Conversely, a study was judged to have a high risk of bias when it reported a feature that would be judged as having a high risk of bias in any domain. As it is not possible to blind either in studies of this nature, we did not assess blinding of participants or personnel for overall risk of bias [23].

### Coding of behaviour change techniques

All intervention procedures were coded using the BCT Taxonomy v1 [19]. Content was coded using the information reported within the methodology sections of identified studies and their protocol papers (where available) to identify the specific BCTs used in each intervention. BCTs targeting SB were coded for the entire intervention and then separately for the computer, mobile and wearable technology components. To minimise bias in interpretation of the tool, a small sample of studies were first assessed by two trained BCT coders (one coder was independent of the research team). Inconsistency in coding was reviewed and a consensus reached, prior to the analysis of the remaining studies, by one author. Where uncertainties later arose, the example was discussed with the wider remaining research team to achieve consensus.

### Measures of treatment effect

Fifteen studies reported continuous outcomes for measures of SB across the same scale allowing meta-analysis of mean differences (MD). Statistical analysis was conducted in accordance with guidelines from the Cochrane Handbook for Systematic Reviews of Interventions [22]. SB data were transformed into minutes per day (e.g. 5 h/day = 300 min/day). Data were pooled to compare the post intervention mean differences and 95% confidence intervals (CIs) in sitting time (min/day) between intervention and comparison groups. Authors of the studies included were contacted by email up to three times for further information where required. Studies where the information was unavailable or that reported units that could not be converted to min/day were not included in the meta-analyses.

Where studies reported multiple follow-up points of the same outcome, data were extracted for subgroup analyses at the following time points: short-term (≤3 months), medium-term (>3 to 6 months), and long-term follow-up (>6 months). In studies where two data sets fell within one of these time points, the longest time point was used for data extraction. Where more than one measure of SB was available, objective data were given priority over subjective or proxy data. If more than one proxy measure of SB was available, the measure most representative of overall SB was given preference. If a study focused on reducing workplace SB, workplace SB data were prioritised over SB in other domains or overall SB. Conversely, where an intervention targeted overall daily SB, full day SB data were used in the analysis. Separate subgroup analyses were run for interventions targeting workplace sitting and overall daily SB for short, medium and long-term follow-up periods. Subgroup analyses were also conducted for objective and subjective outcome measures. Data were assessed for statistical heterogeneity. Values of the I^2^ statistic that were 30% to 60% were considered to represent moderate heterogeneity and 50% to 90% substantial heterogeneity. Studies were pooled using a random effects model where heterogeneity was moderate to substantial; otherwise a fixed effects model was used.

## Results

**Fig. 1:**
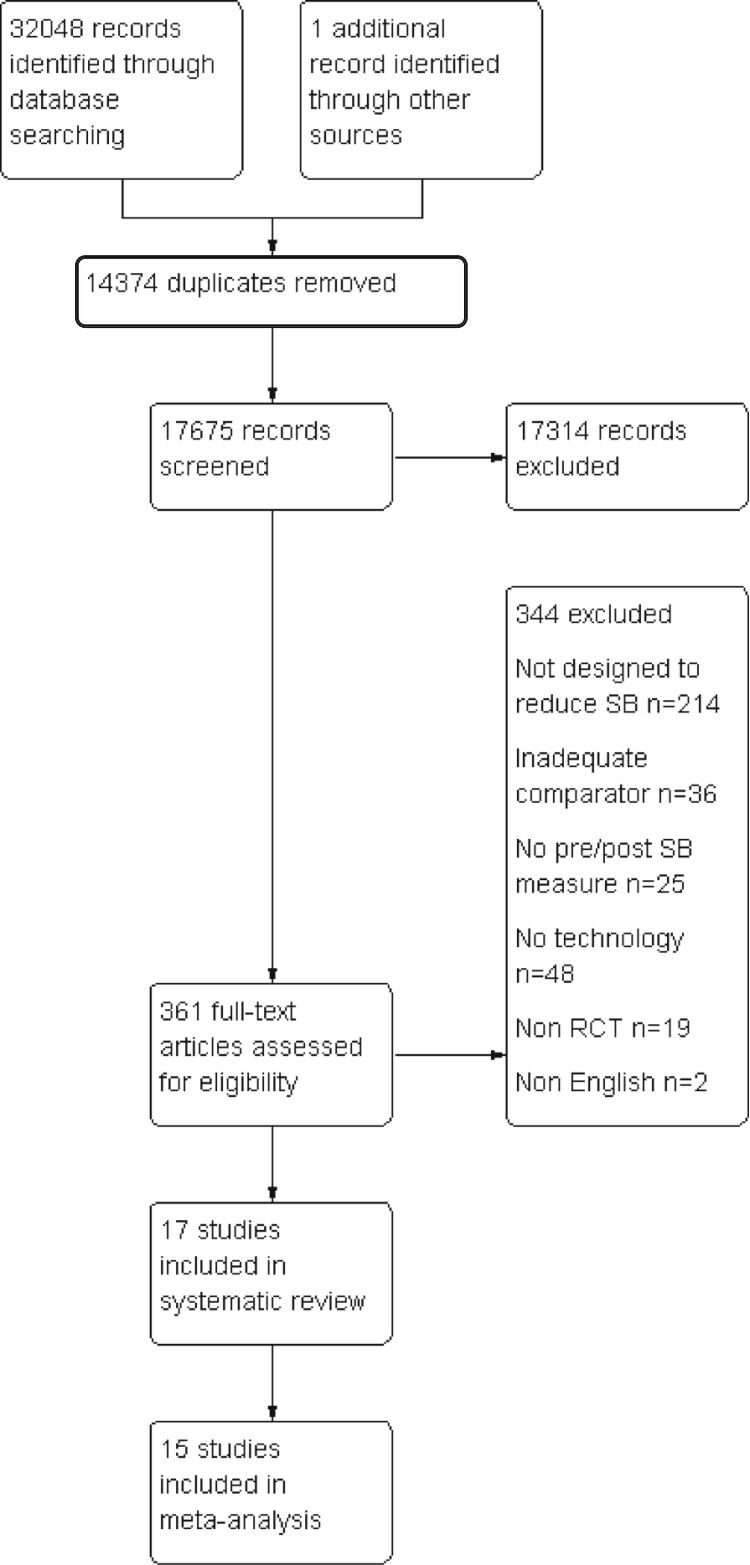
PRISMA flow diagram

Figure 1 displays the PRISMA flow diagram of the literature search. Inclusion criteria were met by 17 studies, 15 of which provided adequate data to be included in a meta-analysis.

### Study characteristics

Study and participant characteristics are summarised in Table 1. Of the 17 included studies (*n* = 1967 participants), 1323 participants (67%) reported being female. Four studies stated the ratio of male to female participants for the sample analysed and not the sample randomised [24–27]. Fifteen studies were carried out in mixed gender populations. Two studies were carried out amongst female participants only [28, 29]. Thirteen studies included any participants aged 18 years or over. One study targeted females aged 55–70 years [29]. The target population in two studies were young adults with an age range of 18–40 years [30, 31]. One study targeted undergraduate university students [25].

**Table 1:** Summary Table of included studies

Author/Year	Study design	Sample Size	Gender	Age	Health Risk	Setting	Aim	Intervention	Technology tool(s)	Comparison group	Outcome Measure	Longest follow-up
Ashe 2015	Pilot RCT	25	25F 0M	All: 64.1 ± 4.6 I: 64.8 ± 4.6 C: 63.1 ± 4.8	Healthy, not meeting PA guidelines	Community/ home	SB + PA	Group health education, PA/SB, PA prescription, online activity monitor (Fitbit), Fitbit app, public transport tickets	Activity monitor (Fitbit) with online companion and app	Health information	ActiGraph™	6 months
Barwais 2013	RCT	33	11F 22M	All:27 ± 4.0 I:9.0 ± 4.4 C:26.4 ± 3.0	Self-reporting >7 h per day sitting	Community/ home	SB + PA	Gruve activity tracker with online companion, motivational emails	Gruve activity tracker with online companion, motivational emails	No intervention	7-day SLIPA Log	4 weeks
Biddle 2015	RCT	187	128F 59M	All:32.8 ± 5.6 I:32.4 ± 5.4 C:33.3 ± 5.8	Obese/overweight plus additional risk factor for diabetes	Community/ home	SB	Group education, Gruve activity tracker with online companion, motivational texts/calls	Gruve activity tracker with online companion, motivational texts	Health information + SB information	ActiGraph™, ActivPAL™, IPAQ, Marshall sitting questionna-ire	12 months
Carr 2013	RCT	40	36F 4M	All:44.7 ± 9.6 I:47.6 ± 9.9 C:42.6 ± 8.9	Apparently healthy, Self-reporting <60 min of moderate to vigorous PA per week, overweight, reporting a minimum of 75% of their work day sitting	Workplace + community/home	SB	Pedal machine, commercial website (Walker Tracker, Portland, Oregon, USA), pedometer, motivational emails	Commercial website (Walker Tracker, Portland, Oregon, USA), motivational emails	Waitlist	StepWatch™	12 weeks
Danquah^a^ 2016	Cluster RCT	317	210F 107M	All:46 ± 10 I:46 ± 10 C:45 ± 11	Employees who sit most of the workday	Workplace	SB	Active meetings, lecture, workshop, educational emails/texts, project website	Emails/text, project website	No intervention	ActiGraph™	12 weeks
De Cocker^a^ 2016	RCT	213	146F 67M	All:40.3 ± 9.1 I:40.5 ± 8.6 C:39.3 ± 9.0	All employees	Workplace	SB	Web based feedback from project website	Web based feedback from project website	Waitlist	ActivPAL™, WSQ	12 weeks
Donath^a^ 2015	RCT	38	23F 8M 7 no data	All:42.42 I:45 ± 12 C:40 ± 10	Free from cardio-vascular disease	Workplace	SB	Standing desks, computer prompts	Computer prompts	Standing desk	ActiGraph™	12 weeks
Dutta^a^ 2014	Cross-over RCT	29	19F 9M 1 no data	All: 40.4 (no SD reported)	Employees who sit most of the workday	Workplace	SB	Standing desks, reminder emails	Reminder emails	No intervention	Modular Signal Recorder 145, Gruve, OSPAQ	4 weeks
Evans^a^ 2012	RCT	30	22F 6M 2 no data	All: 44 I:49 ± 8 C: 39 ± 10	Healthy employees	Workplace	SB	Individual education session, software prompts	Software prompts	Health information + SB information	ActivPAL™	5 days
Judice 2015	Cross-over RCT	10	5F 5M	All:50.4 ± 11.5	BMI < 25.0 kg m − 2; not taking medication, not meeting PA guidelines, free from any major disease	Workplace + community/home	SB	Computer prompts, step monitoring, motivational calls/texts	Computer prompts, motivational texts	No intervention	ActivPAL™	1 week
Laska 2016	RCT	441	298F 143M	All: 22.8 (no SD reported) I:22.9 C:22.8	BMI: 20–34.9 kg/m2	Community/home	Life style + SB	Educational course (online/face to face/hybrid), project website, motivational texts/calls	Educational course (online/ face to face/hybrid), project website, motivational texts	Health information	Self-reported screen time behaviours	24 months
Maher 2015	RCT	195	89F 95M 11 no data	All: 20.4 (no SD reported)	All undergraduates	Community/home	SB	SB planning via email	SB planning via email	No intervention	IPAQ	7 days
Mainsbridge^a^ 2014	RCT	29	24F 5M	All: 40.10 I: 36.73 ± 12.38 C: 42.28 ± 9.59	Employees who sit most of the workday, medically cleared to perform short bouts of PA	Workplace	SB	Group education, prompting software	Prompting software	Health information + SB information	Self-reported sitting	13 weeks
Pedersen^a^ 2014	RCT	34	26F 8M	All: 43 I:41.50 ± 12.39 C: 43.88 ± 9.65	Employees who sit most of the workday, free from existing health conditions	Workplace	SB	Group education, prompting software	Prompting software	Health information + SB information	Self-reported SB	13 weeks
Schuna^a^ 2014	RCT	41	40F 1M	All: 40.1 ± 10.1 I:40.0 ± 9.5 C: 40.3 ± 10.9	Overweight/obese office workers.	Workplace	SB + PA	Treadmill desk, computer prompts	Computer prompts, email	No intervention	ActiGraph™	12 weeks
Urda^a^ 2016	RCT	48	48F 0M	All: 48 ± 10	Employees who sit most of the workday	Workplace	Life style + SB	Educational handout, computer prompt	Computer prompts	No intervention	ActivPAL™	5 days
Van Berkel^a^ 2014	RCT	257	173F 84M	All: 45.5 I: 46.0 ± 9.4 C: 45.1 ± 9.6	All employees	Workplace	Life style + SB	Mindfulness sessions, nutrition support, e-coaching, intranet webpage	E-coaching (email), intranet webpage	Health information	Self-reported SB	12 months

*RCT* randomised controlled trial, *F* female, *M* male, *±* standard deviation, *I* intervention, *C* control, *SB* sedentary behaviour, *PA* physical activity, *7-day SLIPA Log* 7-day Sedentary and Light Intensity Physical Activity Log, *IPAQ* International Physical Activity Questionnaire, *WSQ* Workforce Sitting Questionnaire, *OSPAQ* Occupational Sitting and Physical Activity Questionnaire

^a^denotes interventions targeting workplace sitting

All studies were published between 2012 and 2016. Ten interventions were designed to reduce SB in the workplace and seven interventions aimed to reduce overall daily SB. Eleven studies were SB interventions alone [24–27 30, 32–37], and both PA and SB were targeted in three studies [29, 38, 39]. The remaining three were lifestyle interventions that included a SB reduction component [28, 31, 40].

All studies targeted SB using a mix of intervention approaches. Table 1 details the overall components of the interventions in addition to computer, mobile and wearable technology components. The studies targeting workplace SB utilised the following tools: software/computer prompts were used in seven studies [24, 27, 28, 32, 34, 35, 39]; emails were used in five studies [26, 27, 36, 39, 40]; websites to relay information and provide feedback to participants were used in three studies [36, 37, 40]; and text messages were used in one study [36]. In those interventions targeting overall sitting, emails were used in three studies [25, 33, 38], websites were used in two studies [31, 33] and text messages were sent to participants in three studies [30–32]. Activity monitors with an online companion were used in three studies [29, 30, 38]. One study used a mobile application intervention, and this was an optional component of the intervention [29].

The duration and intensity of the interventions varied. The intervention time ranged from five days [24, 28] to 24 months [31]. The type of control groups also varied between studies. Two studies used a wait-list control [33, 37], seven studies used a no intervention control group [25, 26, 28, 32, 36, 38, 39] and one study compared a stand-up desk combined with prompts with a stand-up desk alone [27]. Seven studies provided their control group with basic health information [24, 29–31, 34, 35, 40].

A variety of SB measurement tools were used. Three studies used more than one measurement tool [26, 30, 37]. Eleven studies used objective measures including; accelerometers [26, 27, 29, 30, 33, 36, 39] and inclinometers [24, 28, 30, 32, 37]. Subjective questionnaires were used in five studies [25, 26, 30, 37, 38]. Four studies used proxy measures where participants were asked to record the time they spent in the domains they were interested in for example computer time, TV time [31, 34, 35, 40].

### Risk of bias of included studies

**Fig. 2:**
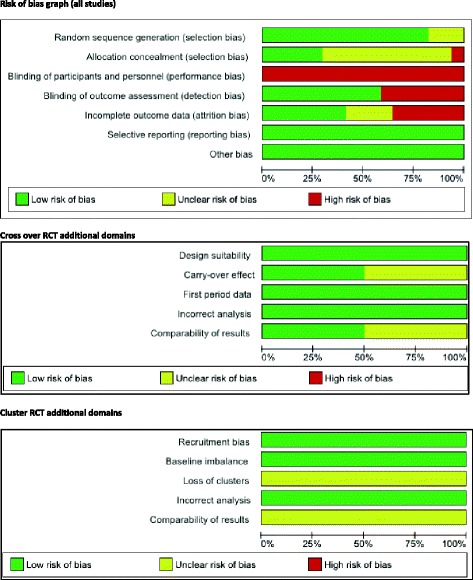
Risk of bias graph

**Fig. 3:**
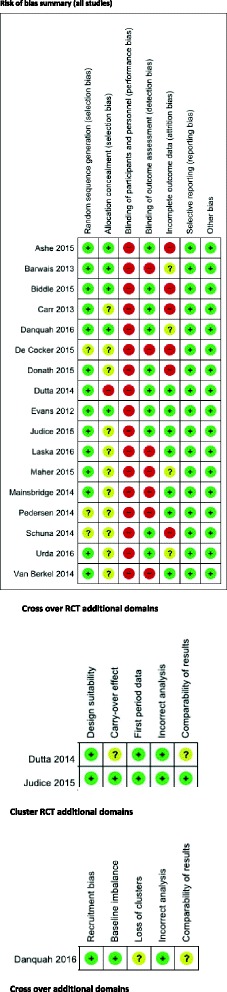
Risk of bias summary

The assessment for each risk of bias item across all included studies, plus the additional domains assessed for cross over and cluster RCTs are presented in Figs. 2 and 3.

### Overall risk of bias assessment

Overall, 13 studies were judged to have a high risk of bias based on: allocation concealment [26] blinding of outcome assessment [25, 31, 34, 35, 37, 38, 40], incomplete outcome data [27, 29, 30, 33, 37, 39]. Three studies were deemed to be at an unclear risk of bias due to incomplete outcome data [28, 36], allocation concealment [28, 32]. One study was judged to be at a low risk of bias [24]. Due to only one study being at low risk of bias, it was not possible to conduct a sensitivity analysis. Refer to Figs. 2 and 3 for a graph and summary of judgements about each risk of bias item for each included study.

### Behaviour change techniques

A total of 104 BCTs were coded in the 17 included studies (Table 2)***.*** 20/93 unique BCTs were coded representing 21.5% of the taxonomy. The range of BCTs coded per study was one to 15. The most frequently coded BCT was “instruction on how to perform a behaviour” which was coded 15 times, “social support (unspecified)” (12 times), “prompts and cues” (11 times) and “adding objects to the environment” (11 times).

**Table 2:** BCT coding and frequency

BCT LABEL	Ashe 2015	Barwais 2013	Biddle 2015	Carr 2013	Danquah 2016^a^	de Cocker 2016^a^	Donath 2015^a^	Dutta 2014^a^	Evans 2012^a^	Judice 2015	Laska 2016	Maher 2015	Mainsbridge 2014^a^	Pedersen 2014^a^	Schuna 2014^a^	Urda 2016^a^	Van Berkel 2014^a^	Total coded (whole intervention)	Total coded (technology)
1. Goals and planning																		25	10
1.1. Goal setting (behaviour)		x	x		x	x					x	x					x	7	5
1.2. Problem solving	xx		xx		x					x	x				x		x	9	2
1.4. Action planning	x		x			x						x						4	2
1.5. Review behaviour goal(s)	x		xx														x	4	1
1.7. Review outcome goal(s)					x													1	0
2. Feedback and monitoring																		10	8
2.2. Feedback on behaviour			x			x												2	1
2.3. Self-monitoring of behaviour		x	x	x						x	x	x	x	x				8	7
3. Social support																		13	8
3.1. Social support (unspecified)	x	x	xxx	x	x					x	xx						xx	12	7
3.2. Social support (practical)															x			1	1
4. Shaping knowledge																		15	4
4.1. Instruction on how to perform the behaviour	xx		x	x	x	x			xx	xx	xx		x	x		x		15	4
5. Natural consequences																		9	1
5.1. Information about health consequences			x		xx	x			xx				x	x		x		9	1
6. Comparison of behaviour																		2	1
6.1. Demonstration of the behaviour														x				1	0
6.2. Social comparison											x							1	1
7. Associations																		11	10
7.1. Prompts/cues			x	x	x		x	x	x	x			x	x	x	x		11	10
8. Repetition and substitution																		6	0
8.2. Behaviour substitution	x				x						x							3	0
8.7. Graded tasks	x				x			x										3	0
9. Comparison of outcomes																		1	1
9.1. Credible source											x							1	1
10. Reward and threat																		0	0
11. Regulation																		0	0
12. Antecedents																		12	3
12.3. Avoidance/reducing exposure to cues for the behaviour											x							1	0
12.5. Adding objects to the environment	xx	x	x	xx	x		x	x		x					x			11	3
13. Identity																		0	0
14. Scheduled consequences																		0	0
15. Self-belief																		1	0
15.1. Verbal persuasion about capability					x													1	0
16. Covert learning																		0	0

*BCT* Behaviour change technique, *x* coded as part of the technology aspect, *x* coded as part of intervention (non-technology aspects)

^a^denotes interventions targeting workplace sitting

A total of 46 BCTs were coded in the 17 studies for the computer, mobile and wearable components of the interventions only. In these interventions, there were 14 unique BCTs coded, ranging from one to 10 per study. The most frequently coded BCTs were “prompts and cues” (10 times), “self-monitoring of behaviour” (7 times), “social support (unspecified)” (7 times) and “goal setting (behaviour)” (5 times).

### Effects of intervention

#### Main analysis

**Fig. 4:**
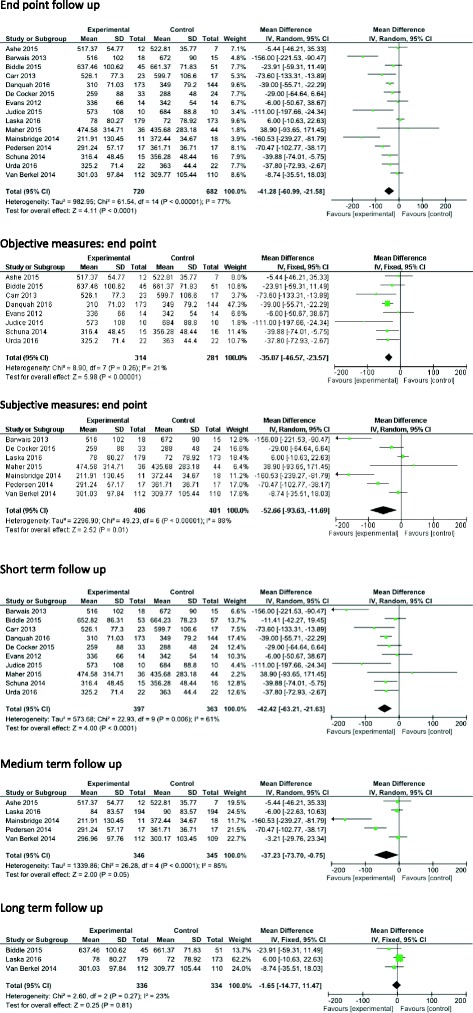
Effects of intervention versus control on SB

Results of the main meta-analysis (*n* = 15; Fig. 4) suggest that SB reducing interventions incorporating computer, mobile and/or wearable technology tools resulted in a mean reduction of −41.28 min/day (95% CI -60.99, −21.58, I^2^ = 77%, *n* = 1402), in the intervention group at end point follow-up.

In the eight studies which reported objective measures of SB [24, 28–30, 32, 33, 36, 39], the pooled analysis resulted in a mean reduction of −35.07 min/day (95% CI -46.57, −23.57, I^2^ = 21%, *n* = 595) in favour of the intervention group. The seven studies which reported subjective measures of SB [25, 31, 34, 35, 37, 38, 40] showed a mean reduction of −52.66 min/day (95% CI, −93.63, −11.69, I^2^ = 88%, *n* = 807).

Ten of the 15 studies included in the meta-analysis reported short-term measures (≤3 months) [24, 25, 28, 30, 32, 33, 36–39]. The pooled analysis showed a mean reduction of −42.42 min/day (95% CI -63.21, −21.63, I^2^ = 61%, *n* = 760) in favour of the intervention group. Five interventions reported medium-term (>3 to 6 months) measures. The pooled effect showed a mean reduction of −37.23 min/day (95% CI -73.70, −0.75, I^2^ = 85%, *n* = 691). Three studies reported long-term measures of SB (>6 months). The pooled analysis showed a mean reduction of −1.65 min/day (95% CI -14.77, 11.47, I^2^ = 23%, *n* = 670).

**Fig. 5:**
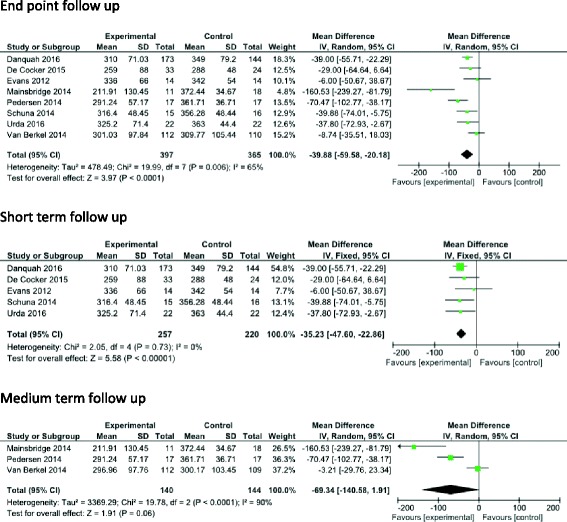
Effects of workplace intervention versus control on workplace SB- short medium and long-term

Eight interventions included in the meta-analysis focused on reducing workplace SB [24, 28, 34–37, 39, 40] (Fig. 5). The pooled effect showed a mean reduction of −39.88 min/workday (time spent at work) (95% CI -59.58, −20.18, I^2^ = 65%, *n* = 762) in favour of the intervention group at end point follow-up.

Five workplace SB studies [24, 28, 36, 37, 39] reported short-term measures, showing a mean reduction of −35.23 min/workday (95% CI -47.60, −22.86, I^2^ = 0%, *n* = 477) in favour of the intervention group. Three workplace SB studies [34, 35, 40] included medium-term measures showing a mean reduction of −69.34 min/workday (95% CI -140.58, 1.91, I2 = 90%, *n* = 284). There were not enough data to conduct a meta-analysis on work place interventions with long-term measures.

**Fig. 6:**
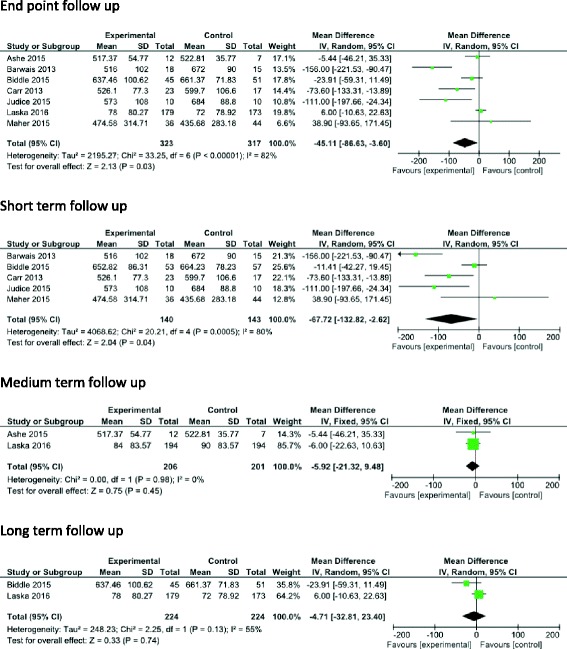
Effects of overall daily SB interventions versus control on daily SB- short, medium and long-term

There were seven interventions targeting overall daily sitting reporting measures of SB [25, 29–33, 38] (Fig. 6). Pooled effects showed a mean reduction of −45.11 min/day (95% CI -86.63, −3.60, I^2^ = 82%, *n* = 640) favouring the intervention group at end point follow-up.

Five of these studies reported short-term measures [25, 30, 32, 33, 38] showing a mean reduction of −67.72 min/day (95% CI -132.82, −2.62, I^2^ = 80%, *n* = 283) in favour of the intervention group. Two studies [29, 31] reported medium-term measures showing a mean reduction of −5.92 min/day (95% CI -21.32, 9.48, I^2^ = 0%, *n* = 413). Two studies [30, 31] reported long-term measures showing a mean reduction of −4.71 min/day (95% CI -32.81, 23.40, I^2^ = 55%, *n* = 448), with substantial heterogeneity in the observed effects studies.

## Discussion

This systematic review and meta-analysis found that SB reduction interventions using computer, mobile and wearable technology resulted in a mean reduction of 41 min/day in the intervention group at end point follow-up. Interventions focusing on workplace SB showed a mean reduction of 40 min/workday in the intervention group at end point follow-up. Interventions focusing on overall daily SB showed a mean reduction of 45 min/day in the intervention group at end point follow-up. Due to risk of bias issues, caution should be taken whilst interpreting these results. Nevertheless, these reductions are encouraging as it has previously been reported that every 30 min of SB reallocated to light PA results in a 2–4% improvement in triglycerides, insulin, beta-cell function biomarkers [41], suggesting clinically meaningful health outcomes.

The magnitude of the mean reduction in sedentary time in this review (41 min/day) is in line with a previous meta-analysis reporting a 42 min/day reduction [9], however, is well below the 91 min/day reduction reported by Prince et al. [8]. This inconsistency may be explained as Prince et al. included non-randomised trials and focused on any intervention that targeted PA and/or SB [8].

The reduction of approximately 40 min/workday in intervention group in this review echoes results from a similar meta-analysis which also showed a reduction of 40 min/workday in favour of the intervention group [42]. Other systematic reviews have shown slightly higher reductions in SB among intervention participants. For example, activity permissive workstation interventions have been reported to contribute to a reduction of 77 min/workday in favour of the intervention group [43]. It is likely that this larger reduction is due to intervention type investigated. These interventions allow participants to stand but also continue working. Although this represents a higher reduction than seen in our review, these work stations are costly to provide and their widespread deployment may not be feasible. From a public health perspective computer, mobile and wearable technology may hold promise for large-scale, cost-effective interventions [17, 44, 45].

The inconsistencies in the above comparisons may be explained by differences in inclusion criteria, as most of these reviews included studies that aimed to increase PA, and/or reduce SB or addressed interventions that reported on SB outcomes, however, did not necessarily target SB in the intervention [8, 9, 17, 20, 23, 42, 46, 47]. This may be relevant as intervention components that successfully increase PA, may not effectively reduce SB, and vice versa [20]. Furthermore, many of the studies in these other reviews were composed of small sample sizes, used different study designs and intervention durations, used a range of SB measurement tools and varying comparator groups. Results from the meta-analysis suggest that SB interventions have the greatest effect on sitting in the short-term, with results lessening over time. Interventions targeting overall daily sitting time also follow this trend. The attenuation of the effects on sitting reported by Martin et al. [9], is similar to that reported in our results, with the greatest impact on SB reduction (42 min/day) in the short-term (≤ 3 months) follow-up declining to 3 min/day at long-term follow-up (>12 months). These results suggest that maintaining long-term behaviour change is challenging, possibly due to the wearing off of the initial “novelty” of technology mediated behaviour change interventions [48, 49]. It must be noted that only three studies reported long-term follow-up measures of SB highlighting a lack of evidence for long-term SB reductions. It was also not possible to analyse interventions targeting workplace sitting at long-term follow-up points as there was insufficient data to conduct a meta-analysis. This lack of long-term evidence is seen in other reviews exploring interventions to reduce SB [42, 46, 50]; where they also did not or could not evaluate long-term effectiveness. Given the importance of sustained behaviour change for health effects, this lack of data highlights the need for studies to examine the effects of longer term SB interventions and over longer follow-up periods.

Greater reductions in SB were found in studies where self-report/proxy measures (53 min/day) of SB were used compared to objective measures (35 min/day). This was also seen in a similar meta-analysis on interventions to reduce SB [9]. This may be due to the subjective assessment of SB being limited by the ubiquitous nature of the behaviours, which may be unremarkable, intermittent and incidental and therefore difficult to accurately recall [51]. Objective measures are also not without limitations. It was not possible to compare cut points and wear time algorithms used in studies, it should be noted that these differences may introduce differences in the scale observed. The development and refinement of valid and reliable objective measures of SB which can incorporate the type and contextual factors, as well as clear guidelines on wear time and cut points are required [52]. This is the first review to collate BCTs used in SB change interventions using computer, mobile or wearable technology in adults. The aim was not to provide definitive conclusions regarding the most effective behaviour change intervention components, but code to identify which techniques have been used to reduce SB. It is, however, difficult to conceptually separate PA promotion and SB reduction components within an intervention [20]. In typical applications of BCT taxonomies in other literatures, a single behaviour is defined and targeted by the intervention, and the link to BCTs can be assumed to be explicitly related to changing that single behaviour [53]. The reality of the design and reporting of many interventions within this review is that they target multiple behaviours and outcomes. Thus, making it more difficult to link BCTs to specific behaviours. Moreover, there was a lack of clear and consistent reporting of which BCTs were undertaken within each intervention making classification of BCTs difficult [54]. Research is warranted to identify the ‘active ingredients’ of successful interventions to refine the design of optimal BCT use and produce an evidence base upon which SB interventions can be developed. In order to assess the impact of BCTs, the reporting of intervention content must be improved. Researchers should “clearly define and provide a rationale for all BCTs that have been included” with full intervention manuals being provided as supplementary electronic files [55]. In complex interventions, clearer delineation of strategies used to change PA and SB, respectively, in intervention reports is required.

The most frequently coded BCTs to reduce SB across the interventions as a whole were “instruction on how to perform a behaviour” “social support (unspecified)”, “prompts and cues” and “adding objects to the environment”. Whereas, the most frequently coded BCTs for computer, mobile and wearable components of the interventions were “prompts and cues”, “self-monitoring of behaviour”, “social support (unspecified)” and “goal setting”. These differences suggest some BCTs may lend themselves well to certain modes of delivery and that the BCTs identified in the technology components might fruitfully be incorporated into future technology based interventions to reduce SB.

When comparing the computer, mobile and wearable components in workplace interventions and overall daily interventions, “prompts and cues” was more frequently coded in workplace interventions and “social support (unspecified)” was more frequently coded in overall daily interventions. This reflects the results in Gardner et al. [20] where it is suggested that workplace SB may be more receptive to planning and routinisation than non-workplace SB, which occurs in less predictable and structured contexts. This highlights the need for interventions to be chosen on the basis of what is most appropriate and feasible in the specific setting [56]. The high usage of the BCT ‘prompts/cues’ identified in this review and that of Direito et al. [17] illustrates that technology may be harnessed to facilitate intervention delivery, however, also to conduct intervention “top-ups” beyond the intervention core duration. This may be a vital component for interventions to prevent relapse.

This study has a number of strengths, including a comprehensive search strategy in multiple databases and the adherence to methodological criteria for high quality-systematic reviews and meta-analysis. In addition, the systematic detailing of BCT coding procedures using the most recent BCT taxonomy (v1), allows future researchers to replicate and review methods used in detail. However, non-English publications were excluded from review and the search was limited to peer reviewed publications. There was considerable heterogeneity of included studies with regard to intervention type, sample size, follow-up duration, type and outcome estimates and no meta-regression was performed. Baseline sitting levels varied across the studies, the scope for change post intervention may be influenced by how much participants sat pre intervention. It must also be noted that how central technology was to each intervention varied greatly. 13/17 included studies were of high risk of bias, with particular concerns in the areas of detection and attrition bias. Six studies were at high risk of attrition bias due to high dropout levels. SB measures used to determine intervention effects in this analysis were measured through subjective measures in seven studies and thus were at high risk for detection bias. These identified methodological flaws present a problem when trying to draw conclusions and evidence presented in the current review should be interpreted with caution. This review also included ‘active’ comparator groups which may contribute to smaller intervention effects. It was not possible to statistically analyse the individual effectiveness of BCTs or to assess the effectiveness of different combinations of behaviour techniques due to the number of different combinations of BCTs present within studies. In order to address this, future study designs could consider using adaptive interventions such as sequential multiple assignment trials (SMART) or multiphase optimization strategy (MOST) designs. Finally, technology development often out-paces academic research [57] and this review includes two studies using the Gruve activity monitor which is no longer commercially available.

This systematic review provides a useful overview of the effectiveness of computer, mobile and wearable technology interventions in reducing SB and has exposed important gaps in the current evidence base which warrant further attention. Future research should focus on attrition rates to reduce drop out and improve engagement. Such studies may consider using technology to refresh the intervention, varying the approach or introduce a new intervention as time passes to encourage long-term maintenance of SB reductions. Furthermore, research should aim to improve detection bias by using objective measurement tools of SB e.g. accelerometer/inclinometer, in order to better detect intervention effects. The lack of long-term follow-up highlights the need for extended follow-up in future studies to examine potential long-term impacts of SB interventions. We also recommend including outcome measures that will be of interest to workplaces and policy makers to determine efficient use of resources such as the cost-effectiveness of technology supported strategies to reduce SB.

## Conclusion

This review provides new knowledge regarding technology interventions incorporating BCTs for reducing SB. Our findings suggest that computer based, mobile and wearable technologies appear to be promising approaches to reduce SB. However, due to risk of bias issues, caution should be taken whilst interpreting these results. The reduction in sitting time appeared to be most prominent at short-term follow-up and attenuated over time, with the exclusion of interventions targeting work place sitting, where results were most prominent at medium-term follow-up. A range of BCTs were implemented in these interventions. Future studies need to improve reporting of BCTs within interventions and address the methodological flaws identified within the review through the use of more rigorously controlled study designs with longer-term follow-ups, objective measures of SB and the incorporation of strategies to reduce attrition.

## Additional files

Additional file 1: PRISMA checklist (DOC 62 kb)

Additional file 2: Search strategy (DOCX 14 kb)

## Abbreviations

7-day SLIPA Log: 7-day Sedentary and Light Intensity Physical Activity Log
AMSTAR: Assessment of multiple systematic reviews
BCT: Behaviour change technique
CI: Confidence interval
Hr/day: Hours per day
IPAQ: International Physical Activity Questionnaire
MD: Mean difference
MET: Metabolic equivalent
Min/day: Minutes per day
NICE: National Institute for Health and Care Excellence
OSPAQ: Occupational Sitting and Physical Activity Questionnaire
PA: Physical activity
PRISMA: Preferred Reporting Items for Systematic reviews and Meta-Analyses
RCT: Randomised-controlled trial
SB: Sedentary Behaviour
WSQ: Workforce Sitting Questionnaire
