# Supplementary material for: Using computer, mobile and wearable technology enhanced interventions to reduce sedentary behaviour: a systematic review and meta-analysis
Source: Int J Behav Nutr Phys Act. 2017 Aug 11;14:105. doi: 10.1186/s12966-017-0561-4 (PMC5553917; doi:10.1186/s12966-017-0561-4)
Supplement: Supplementary file 2 — Search strategy (DOCX 14 kb) [file 12966_2017_561_MOESM2_ESM.docx]

| Medline Search |  |
| --- | --- |
| 1. Internet/ |  |
| 2. Software/ |  |
| 3. Computer Communication Networks/ |  |
| 4. Online Systems/ |  |
| 5. Telemedicine/ |  |
| 6. Mobile Applications/ |  |
| 7. Cell Phones/ |  |
| 8. Smartphone/ |  |
| 9. Computers, Handheld/ |  |
| 10. Text Messaging/ |  |
| 11. Electronic Mail/ |  |
| 12. Reminder Systems/ |  |
| 13. Cues/ |  |
| 14. Wireless Technology/ |  |
| 15. Actigraphy/ |  |
| 16. Accelerometry/ |  |
| 17. (intranet or internet or website$ or web based or computer based or software or online).tw. |  |
| 18. (ehealth or e health or mhealth or m health or electronic health or mobile health or telehealth or tele health or health technolog$).tw. |  |
| 19. (mobile phone$ or smartphone$ or smart phone$ or cellphone$ or cell phone$ or cellular phone$ or hand held or digital device$ or digital technolog$ or mobile technolog$ or mobile device$).tw. |  |
| 20. (mobile app or mobile apps or mobile application$ or mobile phone app or mobile phone apps or mobile phone application$ or smartphone app or smartphone apps or smartphone application$ or smart phone app or smart phone apps or smart phone application$ or phone app or phone apps or phone application$ or cellphone app or cellphone apps or cellphone application$ or cell phone app or cell phone apps or cell phone application$ or tablet app or tablet apps or tablet application$).tw. |  |
| 21. (text messag$ or sms or short message service$ or email$ or e mail$ or electronic mail$ or remind$ or prompt$ or cue$ or cuing).tw. |  |
| 22. (wireless or wearable$ or wristband$ or wrist band$ or wristworn or wrist worn or watch$ or smartwatch$ or smart watch$).tw. |  |
| 23. (activity track$ or activity sens$ or activity monitor$ or movement track$ or movement sens$ or movement monitor$ or just in time adaptive intervention$ or lifelog$ or life log$ or quantified self or self monitoring device$).tw. |  |
| 24. (pedomet$ or acceleromet$ or step count$ or inclinomet$).tw. |  |
| 25. (pervasive technolog$ or pervasive comput$ or ubiquitous technolog$ or ubiquitous comput$).tw. |  |
| 26. Sedentary Lifestyle/ |  |
| 27. (sedentar$ or sitting or seat$ or lying or reclin$ or recumben$).tw. |  |
| 28. (screen time or screentime or computer time or TV time or television time).tw. |  |
| 29. (self track$ or fitness track$).tw. |  |
| 30. 1 or 2 or 3 or 4 or 5 or 6 or 7 or 8 or 9 or 10 or 11 or 12 or 13 or 14 or 15 or 16 or 17 or 18 or 19 or 20 or 21 or 22 or 23 or 24 or 25 or 29 |  |
| 31. 26 or 27 or 28 |  |
| 32. 30 and 31 |  |
